# The Use of Small-Molecule Compounds for Cell Adhesion and Migration in Regenerative Medicine

**DOI:** 10.3390/biomedicines11092507

**Published:** 2023-09-11

**Authors:** Juan Mitchell, Kevin W.-H. Lo

**Affiliations:** 1 School of Dental Medicine, University of Connecticut Health Center, Farmington, CT 06030, USA; juamitchell@uchc.edu; 2 Connecticut Convergence Institute for Translation in Regenerative Engineering, School of Medicine, University of Connecticut Health Center, Farmington, CT 06030, USA; 3 Department of Medicine, Division of Endocrinology, School of Medicine, University of Connecticut Health Center, Farmington, CT 06030, USA; 4 Department of Biomedical Engineering, School of Engineering, University of Connecticut, Storrs, CT 06268, USA; 5 Institute of Materials Science (IMS), School of Engineering, University of Connecticut, Storrs, CT 06269, USA

**Keywords:** cell adhesion, regenerative medicine, tissue regeneration, small molecules

## Abstract

Cell adhesion is essential for cell survival, communication, and regulation, and it is of fundamental importance in the development and maintenance of tissues. Cell adhesion has been widely explored due to its many important roles in the fields of tissue regenerative engineering and cell biology. This is because the mechanical interactions between a cell and its extracellular matrix (ECM) can influence and control cell behavior and function. Currently, biomaterials for regenerative medicine have been heavily investigated as substrates for promoting a cells’ adhesive properties and subsequent proliferation, tissue differentiation, and maturation. Specifically, the manipulation of biomaterial surfaces using ECM coatings such as fibronectin extracted from animal-derived ECM have contributed significantly to tissue regenerative engineering as well as basic cell biology research. Additionally, synthetic and natural bioadhesive agents with pronounced abilities to enhance adhesion in numerous biological components and molecules have also been assessed in the field of tissue regeneration. Research into the use of facilitative bioadhesives has aimed to further optimize the biocompatibility, biodegradability, toxicity levels, and crosslinking duration of bioadhesive materials for improved targeted delivery and tissue repair. However, the restrictive drawbacks of some of these bioadhesive and animal-derived materials include the potential risk of disease transmission, immunogenicity, poor reproducibility, impurities, and instability. Therefore, it is necessary for alternative strategies to be sought out to improve the quality of cell adhesion to biomaterials. One promising strategy involves the use of cell-adhesive small molecules. Small molecules are relatively inexpensive, stable, and low-molecular-weight (<1000 Da) compounds with great potential to serve as efficient alternatives to conventional bioadhesives, ECM proteins, and other derived peptides. Over the past few years, a number of cell adhesive small molecules with the potential for tissue regeneration have been reported. In this review, we discuss the current progress using cell adhesive small molecules to regulate tissue regeneration.

## 1. Introduction

With regard to the biological functioning of many different cell types, cellular adhesion is an essential regulatory mechanism of cell self-renewal and intercellular communication that is also of fundamental importance to the development and maintenance of tissues [1,2,3]. Cellular adhesion has been highlighted as a necessary prerequisite to cell longevity and unimpeded biological activity that effectively precludes apoptotic processes [4,5,6]. For instance, anchorage-dependent cells such as adult stem cells, neurons, osteoblasts, and epithelial cells all utilize cell adhesion as an important prerequisite for sustaining cell niches for subsequent integration, migration, survival, and cell specialization [7,8,9]. On the other hand, late attachment implies that cells have been in the system for longer before adhering to the surface, which could decrease their viability [10].

Cell adhesion plays a crucial role in tissue regeneration by supporting the structural integration of cells, facilitating cell migration and survival, guiding cell differentiation and determination of cell fate, and potentially influencing vascularization processes [4]. Therefore, the ability to regulate and control cell adhesion to biomaterial surfaces is of the utmost importance in the field of tissue regenerative engineering [11,12]. In fact, the ability of cells to rapidly attach to a biomaterial can significantly influence the success of clinical applications involving that biomaterial [13]. Such control requires an understanding of the basic molecular mechanisms underlying the adhesion between cells and their substratum. Two mechanisms of cell adhesion to biomaterials have been proposed: non-receptor-mediated cell adhesion to material surfaces via non-covalent bonding, such as electrostatic interactions, hydrogen bonding, or polar or non-polar interactions, and ionic interactions between various components or molecules on cell membrane and chemical functional groups on biomaterials [14].

Functional receptor-mediated and signal transmitting cell adhesion to a conventional biomaterial is mediated by extracellular matrix (ECM) molecules that are imperative to the in vivo and in vitro survival of adherent cells, such as fibronectin, proteoglycans, integrins, cadherins, vitronectin, elastin, collagen, laminin, and other classes of adhesion molecules [15]. In fact, a growing number of ECM molecules are now implicated in tissue homeostasis, and they are good potential targets for clinical interventions [16]. Tissue regeneration often uses ECM components as a key factor in tissue repair. The ECM provides not only the structural support for cells but also the biochemical cues needed to moderate cell physiology and phenotype [17]. To date, many strategies to control cell adhesion and homing have relied on controlling the placement of ECM proteins on biomaterial surfaces [18]. The most commonly used technique is to immobilize the recombinant ECM protein (i.e., fibronectin) on the biomaterial surface prior to plating the cells [19,20]. However, this is a costly manufacturing process because it requires specific techniques and reagents for immobilization [21]. Moreover, the instability of ECM proteins during immobilization is the most significant obstacle in developing surface-bound ECM biopolymers [22]. 

Another disadvantage of using recombinant ECM proteins is that they may elicit an undesirable immune response in the host [23]. Intriguingly, ECM-derived short peptides like Arg-Gly-Asp (RGD) have been proposed as alternatives for promoting cell adhesion [24]. However, their general lack of specificity and significantly decreased receptor binding affinities have proven detrimental in attempts to regulate the highly specific and integrated processes necessary for tissue regeneration [25,26,27,28,29]. Therefore, alternative strategies are being explored to overcome these limitations and achieve more precise control over the cellular responses involved in effective cell adhesion and tissue regeneration [30,31]. 

Another means of adhesion promotion includes natural and synthetic materials in the form of bioadhesives. In general, bioadhesives are substances that are designed to adhere to biological tissues. Bioadhesives function as especially adhesive compounds on tissues and grafts that amplify graft stability and wound closure [32]. Bioadhesives have been adopted as mediums for localized delivery of cells and growth factors to help facilitate soft tissue adhesion for enhanced wound repair, primarily in musculoskeletal tissues such as bone, cartilage, tendon, and intervertebral discs. However, it should be noted that bioadhesives are not without their own shortcomings that limit their clinical applications. Prominent bioadhesives like fibrin cannot be readily used at defect sites of high-tensile stress and are prone to degradation at accelerated rates [33]. In addition, many other common bioadhesive materials, such as cyanacrylate and PEG-based materials, also lack adequate in vivo analyses on their long-term efficacy and cytotoxicity [34], indicating the need for more thorough research and evaluation. 

The majority of the aforementioned limitations can be overcome using synthetic non-peptidic cell-adhesive small molecules that are capable of promoting cellular adhesion, differentiation, and survival both in vitro and in vivo (see Table 1) [35]. Often classified according to their chemical composition and clinical utility, many small molecules have been established as invaluable drugs with the purpose of preventing or ameliorating common acute and chronic diseases. In fact, small molecules comprise approximately 90% of the pharmaceutical drug market—displaying a fortuitous degree of ubiquity and accessibility. Small molecules exhibit many benefits centered around favorable attributes like nonimmunogenicity, angiogenesis, structural endurance, and low expense, making them prime candidates in the development of novel methods for complex tissue regeneration [36,37,38,39,40,41]. 

In the context of tissue engineering and regenerative medicine, the stability and biodegradation of small molecules can play a significant role. For example, if a small molecule with adhesive properties is integrated into a scaffold, its stability and degradation rate can influence the scaffold’s longevity and functionality. Several classes of small molecules demonstrate ample stability, allowing them to preserve their unique physical and chemical properties over extended periods of time. They also tout a significant degree of biodegradability across many intra- and extracellular environments. Citing such traits, a large number of small molecules have been consistently applied in many biomedical engineering fields, making tangible strides in research on stem cell differentiation and wound healing for severe tissue defects. With this in mind, researchers have directed their attention to the manipulation of intracellular signaling pathways using small molecules as bioactive modulators. The regulatory profiles of these compounds are capable of altering many pathways that are critical to biological growth and development, including but not limited to Sonic Hedgehog (SHH), BMP/Smad, MAPK/ERK, Epac, *β-Runx1*, and RANKL. For instance, preclinical studies have shown that the small molecule strontium ranelate influences bone remodeling pathways by decreasing osteoblast-induced RANKL/OPG synthesis within subchondral bone [42]. 

**Figure 1:**
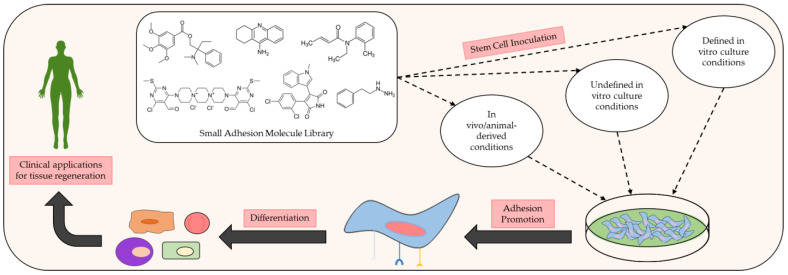
Schematic of the preclinical analysis of small molecules exhibiting beneficial adhesive properties and how they may be used to further develop and improve clinical methods of tissue regeneration. Many small adhesion molecules exert discernible effects on cellular adhesion and differentiation upon inoculation with various stem cell populations under specific in vivo and in vitro conditions. Common in vivo conditions for adhesion assays typically consist of animal models with controlled defects (e.g., injured rats). In vitro conditions for adhesion assays commonly implement defined culture conditions with known amounts of reagent and chemicals in the growth medium or undefined culture conditions in which the exact amounts of reagent and chemicals used in the growth medium are unknown.

A plethora of clinical trials are also being conducted on these molecules to expand their practical portfolio in vivo [43]. Various forms of natural and synthetic scaffolds (e.g., hydrogels, ceramics, fibers, composites, etc.) have been coupled with small molecules to aid in the promotion of osteogenic signaling and sustained release, while also providing a matrix for osteoblast or stem cell adherence at defects sites [44]. Commonly used polymeric scaffolding materials like PLGA, polyurethane, calcium phosphate, and HA microspheres have all exhibited success in the delivery and sustained release of small molecules in vivo. Small molecule incorporation in these scaffolds often entails injection or crosslinking with hydrogels during polymerization, surface modification for external coating with molecules, and other conventional loading methods. Favorable characteristics such as mechanical rigidity, biocompatibility, supplementary adhesive strength, chemical moieties, and surface topography have made matrixial scaffolding an optimal medium for small molecule delivery [45,46]. Specially fabricated scaffolds are also capable of maintaining ECM integrity and avoiding immune-mediated rejection, which could hamper their clinical relevance [47,48,49,50]. With the list of cell adhesive small molecules continuously growing, these light-weight compounds may very well represent the next generation of therapeutics for targeted wound revitalization (see Figure 1). In this review article, we will focus on contemporary advancements in the clinical profile and aptitude of cell adhesive small molecules as they relate to the development of simple, inexpensive, effective, and safe methods for broad tissue regeneration.

## 2. Adhesamine

Recent research has revealed that adhesamine can accelerate the differentiation of hippocampal neurons, mediated by the heparin sulfate-binding mechanism [51]. Adhesamine is the first organic non-peptidic small molecule that has been shown to promote physiological adhesion and growth of cultured cells through surface modulation and selective binding to heparin sulfate on the cell surface [52]. Specifically, it was found that this process involves both the focal adhesion kinase (FAK) and mitogen-activated protein kinase (MAPK) signal transduction pathways [51]. These intercellular pathways are known to link cell surface receptor signals to targets in the cytoplasm or nucleus, promoting activities such as cell migration and morphing, growth, proliferation, and differentiation. FAK plays a primary role in the process of cell adhesion to the extracellular matrix and is intertwined with MAPK. MAPK/ERK [extracellular signal-regulated kinase] kinase (MEK) phosphorylates MAPK, translocating it to the nucleus and allowing it to activate transcription factors that control the expression of genes related to cell growth and differentiation [53]. 

**Figure 2:**
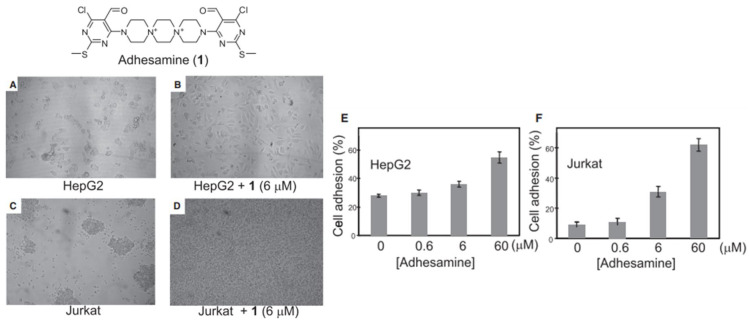
Cell adhesion assay for HepG2 and Jurkat cell lines incubated with 1% (*v*/*v*) DMSO alone or 6 μM of adhesamine (**A**–**D**). Rates of HepG2 cell adhesion to culture plates in varied concentrations of adhesamine, 0–60 μM (**E**). Rates of Jurkat cell adhesion to plates in varied concentrations of adhesamine, 0–60 μM (**F**). Reprinted with permission from Yamazoe et al. [52].

Yamazoe et al. treated human hepatoma (HepG2) cells and Jurkat cells (human T-lymphocytes) with varying concentration of adhesamine (0.6–60 µM) and performed assays for adherence to culture plates following a 3 h or 24 h incubation period [52]. The authors noted that adhesamine enhanced the adhesion of HepG2 cells to the bottom of culture plates by up to two-fold, exhibiting a dose-dependent effect on cellular adhesion with increasing concentrations. Similarly, 30% and 60% of the treated Jurkat cells attached to the culture plates in the presence of 6 mM and 60 mM of adhesamine, respectively (see Figure 2). However, no intercellular adhesion between HepG2 or Jurkat cells within culture plates was observed after the treatment. In their structure–activity relationship studies, six derivatives of the adhesamine molecule with unique modified moieties were fabricated and tested for their individual adhesive effects on Jurkat cells. The six modifications made to the primary adhesamine structure included: substitution of the dispirotripiperazine moiety with an amine linker, dipiperazylethane (molecule 2), methylation of the two nitrogen atoms in the dipiperazylethane linker of molecule 2 (molecule 3), removal of the two terminal pyrimidine rings on the primary adhesamine structure (molecule 4), reduction of the aldehyde groups in the pyrimidine rings to hydroxyl groups (molecule 5), introduction of a fluorescent dansyl group at the C5 position (molecule 6), and introduction of a dansyl conjugate of dispirotripiperazine (molecule 7). Of these derivatives, only molecules 5 and 6 retained their adhesion-promoting activity, while the remaining molecules saw a reduced or complete loss of adhesive qualities. 

**Figure 3:**
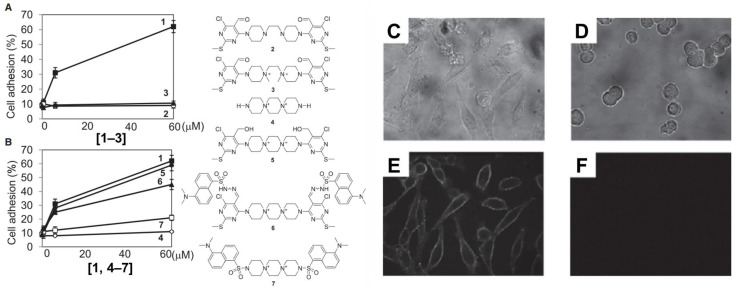
Structure–activity relationship studies surveying the ability of six adhesamine derivatives to promote cell adhesion of Jurkat cells (**A**,**B**). Subcellular localization of fluorescent probes on molecules 6 and 7 using brightfield (**C**,**D**) and confocal (**E**,**F**) imaging analysis for HepG2 cells. HepG2 cells were incubated with heparan degradation enzymes, 6 mM of molecule 6 (**C**,**E**), and 6 mM of molecule 7 (**D**,**F**) for 3 h after seeding. Reprinted with permission from Yamazoe et al. [52].

To further characterize adhesamine’s cellular target sites, the subcellular localization of molecules 6 and 7 in HepG2 cells was determined under fluorescent microscopic observation. For this localization, HepG2 cells were incubated for a 3 h period in media containing 6 mM of molecules 6 or 7 and variants of heparan degradation enzymes. Incorporation of heparinase and heparitinases I and II in the solution medium allowed for verification of the targeting of surface heparan sulfate by adhesamine for cellular adhesion (see Figure 3).

## 3. L1CAM and L1 Agonists or Mimetics

**Figure 4:**
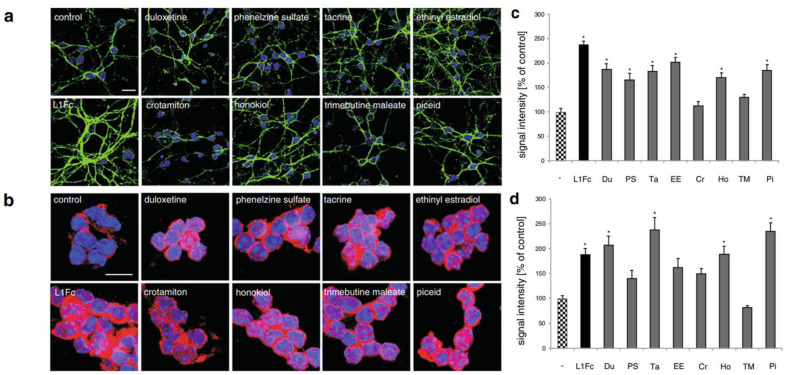
Immunostaining assay displaying the effects of small molecule L1 agonists on the extracellular domain and cytoplasmic expression of L1 protein indicating cell surface L1 and total L1 levels, respectively, in cerebellar neurons. Cells were treated with L1Fc or L1 agonists for 48 h and immunostained with antibody 555 (green) against the extracellular domain of L1 (**a**) or with antibody 172-R against the cytoplasmic domain of L1 (**b**); nuclei were stained with DAPI (blue). Signal quantification of cell surface L1 (**c**) and nuclear L1 (**d**) expression. Statistical significance of difference to untreated control determined at *p* < 0.05; *denotes significance in figure data. Reprinted with permission from Kataria et al. [56].

L1CAM is a transmembrane neuronal cell adhesion molecule within the L1 family with strong implications in cell adhesion, migration, survival, neuritogenesis, synapse formation, and plasticity [54]. The L1 peptide, known to directly facilitate mechanisms of cellular adhesion, has been associated with the activation of multiple signaling pathways. Some researchers have studied the use of molecules to upregulate these L1 pathways and enhance their functions (e.g., Erk). L1CAM is naturally expressed in a plethora of cell types, including but not limited to neurons and Schwann cells [55]. The study by Kataria et al. screened small molecules libraries of known drugs for L1 agonists and evaluated the effects of “hit” compounds via thorough in vitro and in vivo analyses [56]. Specifically, in vitro cell-based assays pinpointed eight “hits” that were identified as small molecule L1 agonists that stimulate neuronal migration, survival, and neurite outgrowth. These included well-established compounds like duloxetine, phenelzine sulfate, tacrine, ethinyl estradiol, crotamiton, honokiol, trimebutine-maleate, and piceid. The authors demonstrated that these small molecular compounds were capable of competitively binding to L1 peptides in the presence of the L1-binding substrate antibody 557, successfully reducing antibody 557-L1–peptide interactions during their molecule screening assay. Immunostaining assays administered using the selected L1 agonists showed that each small molecule tested significantly enhanced L1 expression along the surface and within the cytoplasm of cerebellar neurons. The highest levels of L1 surface expression were observed in neurons treated with duloxetine, phenelzine sulfate, tacrine, ethinyl estradiol, honokiol, and piceid. The highest nuclear L1 levels were observed in neurons treated with duloxetine, tacrine, honokiol, and piceid (see Figure 4). These molecules also supplemented L1-mediated functions such as neuronal outgrowth and cellular migration to the site of injury. In relation to neuron migration, the authors found that piceid and phenelzine sulfate aided migration the most out of all molecules tested. The remaining six molecules also prompted neuron migration to a notably higher extent than the control (143–172% vs. the control group’s 100%). 

In relation to Schwann cell migration, six molecules effectively promoted migration (duloxetine, phenelzine sulfate, piceid, honokiol, ethinyl estradiol, and trimebutine maleate), while the remaining two were determined to be ineffective in promoting migration (tacrine and crotamiton). When assessing L1-activated signaling and phosphorylation events, the authors observed that all the compounds stimulated Erk phosphorylation except for tacrine. The small molecules duloxetine, phenelzine sulfate, and honokiol displayed the strongest stimulation of Erk phosphorylation. The findings of this study have partially illuminated the aptitude of such molecules for repurposing into therapeutic agents that promote tissue revitalization through increased adhesion. Another study with an identical library of L1 agonists and a similar experimental design found that polydatin, a small glycoside, also incited L1-mediated neurite outgrowth along with neuronal migration [57]. Perhaps more importantly, some in vivo studies have revealed that L1 agonist small molecules can discernibly enhance the functional regeneration and re-myelination of neurons in a femoral nerve injury mouse model. Moreover, the L1 agonists also improved recovery of motor functions in a spinal cord injury mouse model. In the in vivo study on young mice with spinal cord injuries conducted by Xu et al., the L1 agonists trimebutine and honokiol were surveyed for their influence on L1 levels following tail vein injection [58]. Six weeks post spinal cord injury, the authors observed a marked increase in the expression of L1, pCK2α (a regulator of endocytic trafficking of L1 and L1-stimulated axon growth), and mTOR (proproliferative) proteins as well as phosphorylation in neurons localized to the spinal cord lesion centers, especially in mice treated with trimebutine. No substantial change in L1 expression was noted for the group treated with honokiol. However, both trimebutine and honokiol were observed to initiate L1-activated signaling cascades within cerebellar granule cells (trimebutine prompting pathway induction at 5 nM and honokiol prompting induction at 50 or 100 nM). While it was inevitably determined that trimebutine had a much more profound impact on axonal regrowth and restoration of locomotor activity than honokiol, both small molecules still enhanced neuronal regeneration in injured mice.

Phenelzine, a small L1 mimetic molecule and monoamine oxidase inhibitor commonly prescribed for its antidepressant effects, has been the subject of many adhesion analyses assessing the quality of cellular adhesion in the presence of varied cell types [59]. Most inquiries into the adhesive properties of phenelzine have been centered around its effect on L1 adhesion molecules [60]. Two studies by Ri et al. found that phenelzine is capable of stimulating functional recovery, remyelination, and neuronal survival in both larval zebrafish and young mice through upregulation of L1.1 protein levels [59,61].

## 4. cAMP-Mediated Adhesion Utilizing Small Molecules

The manipulation of bone cell adhesion to biomaterials is an essential requirement for successful bone regeneration as well as orthopaedic applications. Indeed, cell adhesion to biomaterials is important for influencing subsequent biological processes such as cell survival, osteogenic differentiation, and ultimately mineralization. The cyclic adenosine monophosphate (cAMP) signaling pathway, involving effectors like protein kinase A (PKA), exchange protein activated by cAMP (Epac), and cyclic nucleotide-gated channels, is one of the known signaling mechanisms required for integrin-mediated cell adhesion in many cell types. cAMP is a small, ubiquitous, and dynamic secondary messenger that regulates a variety of cellular processes incumbent to a diverse array of tissue types, including regenerative processes like proliferation, differentiation, angiogenesis, and osteogenesis [62]. Interestingly, Lo et al. demonstrated that osteoprogenitor cell (i.e., MC3T3-E1) adhesion to poly(lactic-co-glycolic acid) (PLGA) based biomaterials can be significantly enhanced through manipulation of cAMP-mediated signaling in vitro by using cAMP-related small molecules such as 8-Bromoadenosine 3′, 5′-cyclic monophosphate (8-Br-cAMP), N⁶-Benzoyladenosine-3′, 5′-cyclic monophosphate (6-Bnz-cAMP), and forskolin. In fact, the authors found that 0.1 mM of 8-Br-cAMP and 6-Bnz-cAMP increased the initial MC3T3-E1 cell adhesion to PLAGA thin films. It was concluded that such adhesion occurred due to 8-Br-cAMP’s and 6-Bnz-cAMP’s induction of PKA signaling mechanisms. Trials with a PKA-inhibitor (H89) confirmed that PKA was the primary guiding pathway for cAMP analogue-induced adhesion. Forskolin, an adenylyl cyclase agonist drug, also exhibited an increase in PLAGA cell adhesion comparable to both cAMP analogues at a 0.1 mM concentration. 

**Figure 5:**
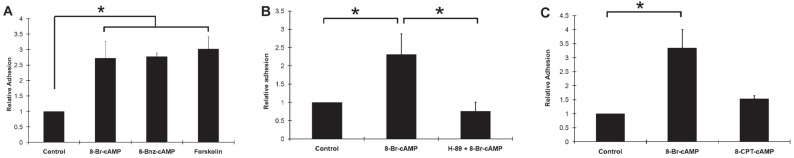
Comparative analysis of cAMP analogs in promoting cell adhesion. Osteoblast-like MC3T3-E1 cells treated with PKA-specific cAMP analog (0.1 mM 6-BnzcAMP), cAMP elevating agent (0.1 mM forskolin), and a positive control (0.1 mM 8-Br-cAMP) (**A**). The effects of PKA inhibition on cell adhesion; MC3T3-E1 cells preincubated with 30 mM PKA inhibitor H-89 and a positive control (0.1 mM 8-Br-cAMP). (**B**). The effects of Epac-specific cAMP analogue on cell adhesion; MC3T3-E1 cells treated with an Epac-specific activator (0.1 mM 8-CPT-cAMP) and a positive control (0.1 mM 8-Br-cAMP) (**C**). Statistical significance determined at *p* < 0.05; * denotes significance in figure data. Reprinted with permission from Lo et al. [63].

The molecule 8-CPT-2Me-cAMP, another cAMP analogue that works solely on Epac signaling pathways, displayed suboptimal levels of cell adhesion during trials compared to the PKA-specific molecules (see Figure 5). The extent of adhesion was characterized using a crystal violet stain assay and hemacytometer for measuring the optical density and cell number, respectively. RGDS peptide competition experiments and serum-starved culture conditions were also administered for 8-Br-cAMP, verifying its action on integrins within the ECM [63]. Additionally, findings from other studies further supported that the cAMP analogues 8-Br-cAMP and 6-Bnz-cAMP partially induced integrin-dependent cell adhesion pathways essential to the osteoblastic differentiation of MC3T3-E1 cells after only one day of inoculation [64,65,66]. One study by Kim et al. verified 8-Br-cAMP’s in vitro and in vivo roles in promoting the adhesion and migration of mouse embryonic stem cells (mESCs) to skin wound sites for stimulated healing in male ICR mice [67]. Intriguingly, the authors also noted that 8-Br-cAMP was capable of disrupting the cellular adherent junction and regulating the actin cytoskeleton of mESCs, encouraging the cellular motility of the assayed stem cells. Moreover, such studies have shown that these cAMP analogues had additional effects on cell differentiation and mineralization in bone tissue.

## 5. Other Identified Adhesive Small Molecules and Small Molecule-Incorporated Scaffolds or Gels

While the adhesive benefits of the aforementioned small molecules have been demonstrated across numerous studies spanning the last two decades, there remain other adhesive molecules of lesser reputation with similar properties of both cellular adhesion and differentiation that warrant further exploration and investigation. 

The small molecule dimethyloxalyglycine (DMOG) is an example of one such proponent of adhesion in MSCs. Adhesion assays conducted by Costa et al. showed that MSC spheroids (comprised of approximately 500 cells) incubated with 500 µM DMOG were more adherent to the bottom of fibronectin-coated well plates. MSC spheroids pre-exposed to DMOG adhered to fibronectin in the well plates at a rate of 69 ± 7%, which was an increase compared to the 49 ± 6% observed in MSC spheroids pre-exposed to hypoxic or normal atmospheric conditions [68]. Similarly, the small molecule GSK-3 inhibitor SB216763, when incorporated into chitosan-based silk nanofiber (CSNF) scaffolds and incubated with human dermal fibroblasts (HDF) for seven days, supplemented cellular adhesion to well plates [69]. A study by Quiros et al. found that the anti-inflammatory small molecule resolving E1 (RvE1) has notable qualities in promoting cellular adhesion in addition to its profound reparative abilities at epithelial wound sites [70]. In migrating human intestinal epithelial cells (hIEC), RvE1 spurred increased ROS signaling and localization of pFAK-Y861 protein within these cells. In an adhesion assay carried out by the authors, the required detachment force needed for separating IECs from the ECM was measured in the presence of RvE1. The IECs seeded on a fibronectin-coated coverslip and left to adhere for 6 h demonstrated greater adherence strength in the RvE1 treatment group (126.9 ± 4.70 dyn/cm^2^) compared to the control group (111.1 ± 1.83 dyn/cm^2^). This denoted the regulatory role of RvE1 in stimulating cell matrix adhesion and IEC migration. 

Spakova et al. showed that the heterocyclic small molecule kartogenin improved the adhesion of human bone marrow-derived mesenchymal stem cells (hBMSCs) to osteoarthritis osteochondral explants [71]. SEM imaging of osteochondral explants showed that hBMSCs cultured with 10 kartogenin displayed higher cell densities than those devoid of kartogenin upon closure of the 21-day culture period. Histological analysis of the cultured hBMSCs further elucidated the pro-adhesive nature of kartogenin, depicting enhanced hBMSC staining (H&E; Safranin O; Alcian blue) along the cartilaginous edge of the explants. 

Employing a somewhat innovative approach to the evaluation of multiple molecules, one group of researchers fabricated and assayed a specially tailored adhesive chemical cocktail [72]. This chemical cocktail was measured for its effects on the endothelialization of stem cells from apical papilla (SCAP) as well as their migration ability following differentiation. In terms of components, the cocktail was comprised of five pro-adhesion small molecules embedded at varying concentrations, including 0.5 μM valproic acid, 3 μM CHIR99021, 1 μM repsox, 10 μM forskolin, and 5 μM Y-27632. After eight days of incubation, the cocktail helped facilitate the differentiation of SCAPs into SCAP endothelial cells (SCAP-ECs) that expressed elevated levels of adhesive endothelial-specific gene proteins like VE-cadherin. Additionally, SPCA-ECs exposed to the cocktail exhibited an identical degree of cell migration to human umbilical vein endothelial cells (HUVECs) in the study’s Transwell migration assay after a 24 h setting period. 

Another molecular target for potentially increasing intercellular adhesion across several tissue types is a member of the cadherin family of cell adhesion molecules, Neuronal (N)-cadherin. N-cadherin as an adhesive molecule that works via homophilic and Ca^2+^-related mechanisms to actively promote migration, outgrowth, and axonal guidance in common cells like neurons. In order to explore the effect of N-cadherin agonist molecules on neurite outgrowth, specifically in the retinal ganglion cell (RGC) of embryonic chicks, Burden-Gulley et al. generated a catalogue of eight peptidomimetic small molecules containing the N-cadherin specific cell adhesion recognition sequence HAVD [73]. These peptidomimetics were separated into a 400 series (molecules ADH-200408, ADH-200433, ADH-200439, and ADH-200442) and 700 series (molecules ADH-200717, ADH-200753, ADH-200786, and ADH-201707) based on the parent compound from which they were derived (either compound 25 or 35 from U.S. patent 7,446,120 B2). The selected parent compounds closely resembled the N-cadherin agonist N-Ac-CHAVDCNH2′s molecular structure, as was desired for this study. Through neurite outgrowth trials, the authors determined that all eight small molecule derivatives (concentrated at 50–200 μM) successfully stimulated N-cadherin-mediated activity, as demonstrated by significant enhancement of RGC neurite outgrowth (129–161% control) despite purposefully suboptimal concentrations of N-cadherin. However, it was noted that only five of the eight peptidomimetic small molecules prompted adhesion-based migration of RGCs on an N-cadherin substrate. These five molecules were also the most potent molecules for stimulating outgrowth in the neurite outgrowth trials (ADH-200408, ADH200433, ADH-200439, ADH-200786, and ADH-201707).

**Figure 6:**
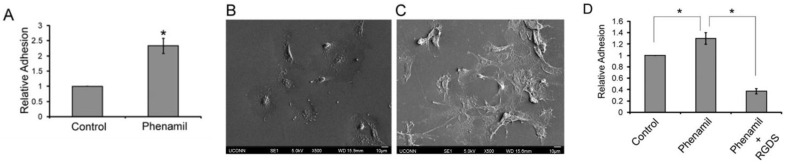
Effects of 10 mM phenamil in osteoblast-like MC3T3-E1 cells on 2D PLGA after a 3 h incubation period (**A**). Untreated MC3T3-E1 cells exhibited a spherical shape with zero, one, or two filapodial extensions (**B**), while phenamil treated MC3T3-E1 cells were attached to 2D PLGA in a flattened cell shape (**C**). Phenamil-stimulated cell adhesion is mediated by integrins. The cells were preincubated with RGDS peptide (0.1 mg/mL) for 20 min in a serum-free condition and then allowed to adhere to 2D PLGA with phenamil for 3 h in the regular growth medium condition (**D**). Statistical significance determined at *p* < 0.05; * denotes significance in figure data. Reprinted with permission from Lo et al. [74].

Numerous studies have also noted the benefit of coupling biocompatible scaffolds and adhesive small molecules in order to further supplement cellular capacity for adhesion. For instance, the pyrazine molecule phenamil—an osteogenic small molecule—has already been reported in the literature for its proliferative and mineralizing properties, yet it has not been subjected to many adhesion assays. However, two studies conducted by Lo et al. aimed to incorporate phenamil into 2D PLGA thin-film scaffolds for the evaluation of MC3T3-E1 cell adhesion and osteodifferentiation [74,75]. These studies found that phenamil-loaded PLGA thin films prompted a greater than two-fold increase in MC3T3-E1 cell adhesion to scaffolds after a 3 h incubation period and were associated with focal adhesion kinase (FAK) and cyclic AMP response element-binding protein (CREB) activation. The adhered cells were noted to exhibit a more flattened or spherical shape than the non-adhered cells. Peptide competition experiments using phenamil were also conducted, which showed that the presence of integrin-binding RGDS peptide in cells impeded phenamil-induced cell adhesion to the PLGA thin films (see Figure 6). Therefore, the prospect of phenamil-mediated cellular adhesion through integrin-based activity was further supported. Another study employed a PLGA microsphere/CECM composite scaffold embedded with kartogenin to assess the adhesion profile of BMSCs following a 48 h cell culture period. The SEM imaging conducted for these composite scaffolds demonstrated amplified adhesion of BMSCs to the scaffold surfaces [76].

## 6. Concluding Remarks and Future Directions

**Figure 7:**
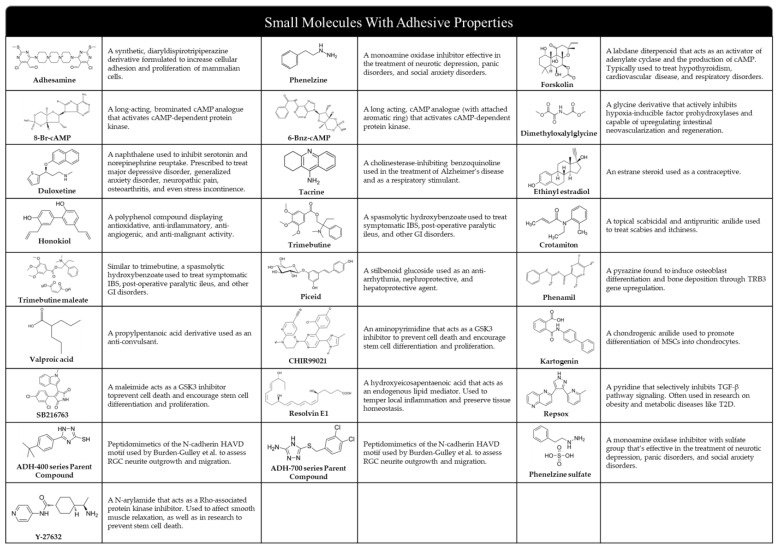
Molecular structures and conventional applications of small molecules that have been investigated for their pro-adhesive properties.

Throughout this review, we identified several adhesive small molecules that have undergone thorough analysis and summarized their purported adhesive benefits as reported across several relevant articles (see Figure 7). We found that across many of the in vitro and in vivo studies exploring the adhesion properties of such molecular agents, there were a number of advantages to cellular adhesion provided by the use of certain small molecules (see Table 2 and Table 3). One resounding benefit of employing the adhesive properties of small molecules is the development a proactive approach to minimizing cytotoxic side effects from therapeutics. Small molecules can curtail some of these cytotoxic effects by limiting the time of drug exposure at the target sites. Traditionally, bone regenerative methods often require long-term continuous exposure to a supra-physiological dosage of therapeutics. It should be noted that therapeutic agents, when used with a large quantity in the long-term, might result in adverse effects. Therefore, the short-term exposure of cells with cell adhesion molecules, together with the drug delivery method utilizing biodegradable polymeric scaffolds, would provide a very promising strategy to mitigate the negative effects associated with therapeutics. This innovation will certainly have a significant impact on the clinical translation of small molecule-based therapeutic strategies by addressing the major concerns associated with it [77]. The exploration of novel small molecules with adhesive properties could lead to the development of advanced bone scaffolds. Adhesive small molecules have the potential to revolutionize tissue engineering by improving cell–scaffold interactions, leading to better regeneration outcomes. Future research in this area is important for the advancement of effective and innovative regeneration strategies.

It is also worth noting that a one-step surgical procedure has been proposed by Jurgens et al. in which adipose tissue-derived mesenchymal stem cells are harvested, seeded on a scaffold, and re-implanted during the same surgical procedure to facilitate bone and cartilage tissue engineering [78]. Using a similar approach, we focused on triggering stem cell such as ADSC toward bone lineage-specific differentiation, with short exposure to adhesive small molecules and implantation of the resulting regenerative-engineered construct in an established pre-clinical orthopedic model. Thus, the goal is to validate the therapeutic treatments, ADSC seeding steps, and delivery methodology to determine their feasibility and ensure they can be integrated into a one-step surgical procedure within the designated time frame. That said, while the clinical significance of these small molecular compounds has yet to be completely realized through medical integration and translation, lab groups procuring small molecules will continue to administer clinical trials that expound upon the adhesive properties of these compounds and convert them into accessible products for improved patient outcomes.

## Figures and Tables

**Table 1:** Comparison of the purported advantages and disadvantages of tissue regenerative proteins/peptides and small molecules.

Molecule Type	Advantages	Disadvantages	References
Proteins	Tissue specificity	Large size; easily cleared from the body; immunogenic; unstable; difficult to fabricate; expensive; can have off- target effects	[18,19,20,21,22,23,24,25,26,27,28,29,30,31,32,33,34]
Short Peptides	Small size; stable; easy to manufacture; chain length can be altered for different applications	Tendency to aggregate; unstable; relatively low affinity to target tissue	[18,19,20,21,22,23,24,25,26,27,28,29,30,31,32,33,34]
Small molecules (<1000 Da)	Inexpensive; non-immunogenic; stable; ability to conjugate easily at high densities; high oral bioavailability; many are FDA-approved for other therapies	No distinguishable deficits detetermined thus far	[35,36,37,38,39,40,41,42,43]

**Table 2:** Small molecules with advantageous adhesive properties in vitro.

Molecule	Classification	Molecular Weight	Working Concentrations	Cell Lineage/Surgical Model	Delivery Methods	Adhesive Properties	References
Adhesamine	Diaryldispirotripiperazine derivatives	670.5 g/mol	0.6–60 µM	HepG2 cells; Jurkat cells	Inoculation in culture plates treated with adhesamine	Enhanced adhesion of HepG2 and Jurkat cells to culture plate by up to 2 fold; adhesive effect is dose dependent	[52]
Phenelzine sulfate	Monoamine oxidase inhibitors (MAOIs)	234.3 g/mol	0.1–100 µM	Cerebellar neurons and Schwann cells isolated from mice	Incubated in well plates treated with phenelzine sulfate	Increased surface and nuclear expression of L1 in cerebellar neurons; enhanced cerebellar neuron and Schwan cell migration	[56]
Forskolin	Labdane diterpenoids	410.5 g/mol	0.1 mM	Osteoblast-like MC3T3-E1 cells	Inoculation in culture plates treated with growth media containing Forskolin	Induced cAMP-mediated cell adhesion of MC3T3-E1 cells to PLAGA thin films	[63]
8-Br-cAMP	cAMP analogues	408.1 g/mol	100 µM	Osteoblast-like MC3T3-E1 cells	Inoculation in culture plates treated with 8-Br-cAMP	Promoted integrin-dependent cell adhesion of MC3T3-E1 cells	[64,71]
			0.02 mM, 0.1 mM, and 0.5 mM	Osteoblast-like MC3T3-E1 cells	Increasing dosages of 8-Br-cAMP introduced to trypsinized MC3T3-E1 cells suspensed in basal medium	Promoted integrin-dependent cell adhesion of MC3T3-E1 cells to PLAGA thin films	[63]
			10 µM	mESCs	Incubated in laminin-coated well plates treated with 8-Br-cAMP	Evoked substantial migration of cells into the denuded areas; induced the translocation of junctional proteins from the plasma membrane to the cytosol;	[67]
6-Bnz-cAMP	cAMP analogues	455.3 g/mol	100 µM	Osteoblast-like MC3T3-E1 cells	Inoculation in culture plates treated with growth media containing 6-Bnz-cAMP	Promoted integrin-dependent cell adhesion of MC3T3-E1 cells	[64,65,66]
			0.1 mM	Osteoblast-like MC3T3-E1 cells	Introduced to trypsinized MC3T3-E1 cells suspensed in basal medium	Promoted integrin-dependent cell adhesion of MC3T3-E1 cells to PLAGA thin films	[63]
Dimethyloxalylglycine	Glycine derivatives	175.1 g/mol	500 µM	MSC spheroids	Incubated in fibronectin-coated well plates with DMOG medium	69 ± 7% MSC spheroids pre-exposed to DMOG adhered to fibronectin in the well plates, an increase from the 49 ± 6% MSC spheroids pre-exposed to hypoxic or normal atmospheric conditions	[68]
Duloxetine	Naphthalenes	297.4 g/mol	0.1–100 µM	Cerebellar neurons and Schwann cells isolated from mice	Incubated in well plates treated with duloxetine	Increased surface and nuclear expression of L1 in cerebellar neurons; enhanced cerebellar neuron and Schwan cell migration	[56,57]
Tacrine	Benzoquinolines	198.3 g/mol	0.1–100 µM	Cerebellar neurons and Schwann cells isolated from mice	Incubated in well plates treated with tacrine	Increased surface and nuclear expression of L1 in cerebellar neurons; enhanced cerebellar neuron migration	[56,57]
Ethinyl estradiol	Estrane steroids	296.4 g/mol	0.1–100 µM	Cerebellar neurons and Schwann cells isolated from mice	Incubated in well plates treated with ethinyl estradiol	Increased surface and nuclear expression of L1 in cerebellar neurons; enhanced cerebellar neuron and Schwan cell migration	[56,57]
Crotamiton	Anilides	203.3 g/mol	0.1–100 µM	Cerebellar neurons and Schwann cells isolated from mice	Incubated in well plates treated with crotamiton	Increased surface and nuclear expression of L1 in cerebellar neurons; enhanced cerebellar neuron migration	[56,57]
Honokiol	Phenols	266.3 g/mol	0.1–100 µM	Cerebellar neurons and Schwann cells isolated from mice	Incubated in well plates treated with honokiol	Increased surface and nuclear expression of L1 in cerebellar neurons; enhanced cerebellar neuron and Schwan cell migration	[56]
			50 nM, 100 nM, 200 nM	Cerebellar granule cells isolated from mice at postnatal day 7	Inoculation in culture plates treated with honokiol	Induced L1-mediated intracellular pathway at 50 or 100 nM	[57,58]
Trimebutine	Hydroxybenzoates	387.5 g/mol	5 nM, 10 nM, 20 nM	Cerebellar granule cells isolated from mice at postnatal day 7	Inoculation in culture plates treated with trimebutine	Induced L1-mediated intracellular pathway at 5 nM	[57,58]
Trimebutine maleate	Hydroxybenzoates	503.5 g/mol	0.1–100 µM	Cerebellar neurons and Schwann cells isolated from mice	Incubated in well plates treated with trimebutine maleate	Increased surface and nuclear expression of L1 in cerebellar neurons; enhanced cerebellar neuron and Schwan cell migration	[56,57]
Piceid	Stilbenoid glucosides	390.4 g/mol	0.1–100 µM	Cerebellar neurons and Schwann cells isolated from mice	Incubated in well plates treated with piceid	Increased surface and nuclear expression of L1 in cerebellar neurons; enhanced cerebellar neuron and Schwan cell migration	[56]
			0.01–1000 nM	Cerebellar granule neurons	-	Promoted neurite outgrowth; enhanced cerebellar neuron migration	[57]
Phenamil	Pyrazines	305.7 g/mol	10 µM	Osteoblast-like MC3T3-E1 cells	Solubilized and loaded into PLAGA scaffolds and introduced to well plates with MC3T3-E1 cells	Promoted a greater than two-fold increase in initial cell adhesion	[74]
			10 µM	Osteoblast-like MC3T3-E1 cells	Solubilized and loaded into PLAGA scaffolds and introduced to well plates with MC3T3-E1 cells	Upregulated phosphorylated (pCREB) and p125FAK proteins; promoted integrin-dependent cell adhesion of MC3T3-E1 cells to PLAGA thin films	[75]
SB216763	Maleimides	371.2 g/mol	1–50 ppm	HDF cells	Solubilized and loaded into CSNF-SB bionic composite scaffolds and introduced to well plates with HDF cells	Stimulated HDF adhesion to well plates	[69]
Chemical cocktail *	Assortment (see footnote)	Assortment (see footnote)	Assortment (see footnote)	SCAP-ECs	Incubated on well plates containing culture medium treated chemical cocktail	Stimulated differentiation of SCAPs to SCAP-ECs and helped facilitate migration; upregulated expression of VE-Cadherin	[72]
ADH-400 series ^†^ ADH-700 series ^†^	Peptidomimetics of the N-cadherin HAVD motif	400 series parent: 233.3 g/mol 700 series parent: 275.16 g/mol	50 µM, 100 µM, 200 µM	RGCs	Retinal explants cultured with peptidomimetics of both ADH parent compounds suspended in DMSO	All peptidomimetics enhanced RGC neurite outgrowth (129–161% control); five peptidomimetics prompted RGC migration on a N-cadherin substrate (ADH-200408, ADH200433, ADH-200439, ADH-200786, ADH-201707)	[73]
Resolvin E1	Hydroxyeicosapentaenoic acids	350.4 g/mol	10–500 nM	hIECs (line SKOC15)	Incubated on fibronectin-coated well glass coverslips treated with RvE1	Significantly increased cell adhesion strength to ECM when compared to untreated control (111.1 ± 1.83 control vs. 126.9 ± 4.70 RvE1)	[70]
Kartogenin	Anilides	317.3 g/mol	10 µM	hBMSCs	Co-cultured on osteochondral explants treated with kartogenin	Higher density of adhered hBMSCs on cartilage surface of osteochondral explants when compared to control; enhanced staining of hBMSCs on cartilaginous edge in kartogenin treatment group	[71]
			Not reported	BMSCs	Kartogenin-encapsulated PLAGA microspheres on CECM scaffold	Amplified adhesion of BMSCs to composite scaffold surface	[76]

* Valproic acid (Propylpentanoic acid derivative; 144.2 g/mol; 0.5 mM), CHIR99021 (Aminopyrimidines; 465.3 g/mol; 3 µM), Repsox (Pyridines; 287.3 g/mol;1 μM), Forskolin (Labdane diterpenoids; 410.5 g/mol; 10 µM), Y-27632 (N-arylamides; 247.3 g/mol; 5 μM). ^†^ ADH-200408; ADH-200433; ADH-200439; ADH-200442; ADH-200717; ADH-200753; ADH-200786; ADH-201707.

**Table 3:** Small molecules with advantageous adhesive properties in vivo.

Molecule	Classification	Molecular Weight	Working Concentrations	Cell Lineage/Surgical Model	Delivery Methods	Adhesive Properties	References
Honokiol	Phenols	266.3 g/mol	1 mg/kg	3 month old female mice with SCI	Injection through the tail vein of anesthetized mice	Elevated neuronal levels of L1, pCK2α and mTOR expression and phosphorylation	[58]
Trimebutine	Hydroxybenzoates	387.5 g/mol	1 mg/kg	3 month old female mice with SCI	Injection through the tail vein of anesthetized mice	No substantial change in neuronal L1 expression	[58]
Phenelzine	Monoamine oxidase inhibitors (MAOIs)	136.2 g/mol	500 nM	Zebrafish larvae with SCI	Inoculated in well plates with E3 medium conatining phenelzine	Stimulated L1.1 protein levels for axonal regrowth in zebrafish larvae	[59]
			6 and 12 mg/kg	4–5 month old female mice with SCI	Intraperitoneal injection once daily starting immediately following trauma until 6 weeks after SCI	Stimulated L1.1 protein levels for axonal regrowth in young mice	[61]
